# Impact of human papillomavirus (HPV) self-collection on subsequent cervical cancer screening completion among under-screened US women: MyBodyMyTest-3 protocol for a randomized controlled trial

**DOI:** 10.1186/s13063-019-3959-2

**Published:** 2019-12-27

**Authors:** Lisa P. Spees, Andrea C. Des Marais, Stephanie B. Wheeler, Michael G. Hudgens, Sarah Doughty, Noel T. Brewer, Jennifer S. Smith

**Affiliations:** 1 Department of Health Policy and Management, Gillings School of Global Public Health, University of North Carolina, 1102-G McGavran-Greenberg, CB7411, Chapel Hill, NC 27599-7411 USA; 2 Lineberger Comprehensive Cancer Center, University of North Carolina, Chapel Hill, NC USA; 3 Department of Epidemiology, Gillings School of Global Public Health, University of North Carolina, 2103 McGavran-Greenberg, CB7435, Chapel Hill, NC 27599-7435 USA; 4 Department of Biostatistics, Gillings School of Global Public Health, University of North Carolina, Chapel Hill, NC USA; 5 Department of Health Behavior, Gillings School of Global Public Health, University of North Carolina, Chapel Hill, NC USA

**Keywords:** Cervical cancer, Cancer screening, Human papillomavirus, Under-screened populations, Health disparities

## Abstract

**Background:**

Screening substantially reduces cervical cancer incidence and mortality. More than half of invasive cervical cancers are attributable to infrequent screening or not screening at all. The current study, My Body My Test (MBMT), evaluates the impact of mailed kits for self-collection of samples for human papillomavirus (HPV) testing on completion of cervical cancer screening in low-income, North Carolina women overdue for cervical cancer screening.

**Methods/design:**

The study will enroll at least 510 US women aged 25–64 years who report no Pap test in the last 4 years and no HPV test in the last 6 years. We will randomize participants to an intervention or control arm. The intervention arm will receive kits to self-collect a sample at home and mail it for HPV testing. In both the intervention and control arms, participants will receive assistance in scheduling an appointment for screening in clinic. Study staff will deliver HPV self-collection results by phone and assist in scheduling participants for screening in clinic. The primary outcome is completion of cervical cancer screening. Specifically, completion of screening will be defined as screening in clinic or receipt of negative HPV self-collection results. Women with HPV-negative self-collection results will be considered screening-complete. All other participants will be considered screening-complete if they obtain co-testing or Pap test screening at a study-affiliated institution or other clinic. We will assess whether the self-collection intervention influences participants’ perceived risk of cervical cancer and whether perceived risk mediates the relationship between HPV self-collection results and subsequent screening in clinic. We also will estimate the incremental cost per woman screened of offering at-home HPV self-collection kits with scheduling assistance as compared to offering scheduling assistance alone.

**Discussion:**

If mailed self-collection of samples for HPV testing is an effective strategy for increasing cervical cancer screening among women overdue for screening, this method has the potential to reduce cervical cancer incidence and mortality in medically underserved women at higher risk of developing cervical cancer.

**Trial registration:**

ClinicalTrials.gov NCT02651883, Registered on 11 January 2016.

## Background

More than half of invasive cervical cancers are attributable to screening infrequently or not at all [1]. Almost one fifth of screening-eligible US women report not having completed a Papanicolaou (Pap) test alone within the last 3 years, meaning that they are overdue by current national guidelines [2, 3]. Minority status and low socioeconomic status are associated with lower screening rates, partly due to women’s inability to access preventive care due to resource-related barriers (e.g., poor access to services, inadequate transportation) [4]. Not surprisingly, cervical cancer disproportionately affects low-income and minority populations. Women living below the federal poverty level (FPL) are four times as likely to die from cervical cancer than women living at or above the FPL [5], and black women are more than twice as likely to die from this preventable cancer than white women [6].

Reviews from the Community Preventive Services Task Force (CPSTF) and Cochrane Collaboration recommend client reminders as an effective method for increasing cervical cancer screening in clinic among both regularly screened and under-screened women [7, 8]. Client reminders are sent via mail or telephone to individuals reminding them to attend screening. These reminders often also include information on the benefits of screening, cervical cancer risk, and appointment scheduling assistance [7]. Randomized controlled trials (RCTs) and population-level studies from the USA and Sweden show that offering client reminders with appointment scheduling assistance is effective for increasing screening rates in clinic compared to usual care (i.e., no reminders or appointment scheduling assistance) [9–12]. However, strategies such as mail or phone call reminders have been shown to be less effective or ineffective in increasing cervical cancer screening rates among women who have to take time off work to attend an appointment, have limited transportation options, feel physical discomfort or embarrassment with pelvic examinations, or are not actively participating in a healthcare system [4, 11, 13].

Testing for oncogenic, human papillomavirus (HPV infection), the primary causal agent in cervical cancer, using samples collected by women themselves, yields a similar sensitivity and specificity to HPV testing of physician-collected samples for the detection of high-grade cervical precancerous lesions (cervical intraepithelial neoplasia lesions grade 2 or higher (CIN2+)) [14, 15]. Conducting cervical cancer screening by self-collection of samples at home and return by mail for HPV testing may reduce some of the screening barriers faced by medically underserved women [16]. Consistent with HPV self-collection studies conducted in other states [17–21] and countries [22–29], our two previous studies in North Carolina demonstrated high acceptability of mailed self-collection samples for HPV testing among low-income women overdue for cervical cancer screening (e.g., 96–98% of participants reported being willing to perform HPV self-collection in the future) [30, 31].

Behavioral theory and empirical evidence indicate that lower levels of perceived barriers and higher levels of perceived risk of developing cancer are associated with higher rates of screening completion [32–37]. Several studies in Europe and Canada have found that mailing at-home HPV self-collection kits to women who have not responded to previous cervical cancer screening reminders leads to higher screening completion [15]. For example, in the Netherlands, 31% of under-screened women randomized to self-collection completed screening, compared to only 7% of those who were sent reminders to attend screening in clinic [38]. These studies also identified high rates of follow up to in-clinic Pap testing (up to 95%) among women receiving HPV-positive self-collection results [38, 39] and that use of HPV self-collection at home led to greater detection of CIN2+ than either screening reminders or scheduling assistance [38, 40–42]. These studies were conducted in countries with national screening registries, screening reminder systems, and universal health care, creating a different healthcare landscape than in the USA. To our knowledge, the US studies published to date that have assessed screening completion from HPV self-collection have used self-collection as their primary endpoint, independent of follow up to screening in clinic among self-test, HPV-positive women [17, 43].

### Objectives

In this RCT we will determine whether offering at-home self-collection of samples for HPV testing (followed by assistance scheduling screening in clinic for those with a positive HPV self-test result) increases the completion of cervical cancer screening among under-screened women compared to offering clinic scheduling assistance alone. We also will examine possible mechanisms explaining the effect or lack of effect of the intervention and will estimate the incremental cost per woman screened of offering self-collection of samples at home for HPV testing, with scheduling assistance, compared to clinic scheduling assistance alone. We hypothesize that offering HPV testing by mailing samples self-collected at home will result in greater screening completion, due to an increase in participants’ perceived risk of developing cancer.

## Methods/design

### Overview

We will conduct an open-label, parallel-group RCT of the impact of mailing self-collection samples for HPV testing on cervical cancer screening among women who are rarely or never screened.

### Participants and setting

Participants will include a minimum of 510 women in North Carolina who are overdue for cervical cancer screening (“under-screened”), defined as not having a Pap test in the past 4 years and without HPV testing in the past 6 years. Our definition of being overdue for screening is based on the American College of Obstetricians and Gynecologists screening guidelines, which recommend that women who have a Pap/HPV co-test be screened every 5 years and women who have only a Pap test be screened every 3 years [44]. Since the USA does not have a national screening registry, we will rely on participant self-report to identify women overdue for cervical cancer screening. Participants will be between the ages of 25 and 64 years, as HPV testing has been approved by the Food and Drug Administration (FDA) for primary screening of women age 25 years or older, and uninsured women up to age 64 years may be covered by Medicaid or the North Carolina Breast and Cervical Cancer Control Program (NC BCCCP) [3]. Participants must be non-pregnant, with an intact cervix (no history of hysterectomy), with income ≤ 250% of the US FPL, uninsured or enrolled in Medicaid, and living within the catchment area of a study-associated clinic. Income, insurance, and study eligibility criteria ensure that participating women will be eligible to receive free or low-cost screening and follow-up care through Medicaid or NC BCCCP.

We will recruit participants from 21 counties in North Carolina over an estimated 36 months. Study clinics will be located in 12 of these counties: Bertie, Camden, Chowan, Cumberland, Currituck, Gates, Guilford, Mecklenburg, Pasquotank, Perquimans, Sampson, and Wake. Participants will be recruited through multiple avenues. The primary mode of recruitment will be advertising through printed materials (flyers, posters, roadside signs, door hangers, buses, and newspapers), online (primarily on Craigslist.org), and radio. We will develop comprehensive lists of local agencies and community organizations providing services to low-income women in the target counties and collaborating with these groups to disseminate information about the study to their clients. Study outreach workers will also recruit participants directly at community events and through community organizations.

### Eligibility screening, consent, and enrollment

Women who call the MBMT-3 study phone number, a toll-free study hotline run by the American Sexual Health Association (ASHA), will reach a staff member who will screen callers for eligibility. Additionally, the study website will have a short online eligibility questionnaire; study staff will review the information and follow up on potentially eligible women. Women recruited in person by study staff will also complete eligibility screening verbally at that time.

At enrollment, we will collect contact information (e.g., phone numbers, best call times, mailing addresses, email, and an alternate contact if the participant cannot be reached directly) to provide multiple options for follow up. Eligible women will receive informed consent forms by mail, along with Health Insurance Portability and Accountability Act (HIPAA) authorization forms to obtain screening results and treatment outcomes from collaborating clinics and outside providers, if needed.

### Randomization and blinding

When the study receives eligible women’s completed consent forms, they will be enrolled into the study and randomized to the intervention or control arm. Randomization will be by permuted block design using blocks of 9 (due to the planned 2:1 ratio for the ratio of intervention to control) [45], stratified by county, using the randomization function of the web programming language hypertext processor (PHP) [46]. We will have a larger intervention group to support analyses of the responses of women with self-collection HPV-positive results. Neither study participants nor study staff will be blinded to the assigned arm. Interviewers will be blinded to study arm for the portion of the post-intervention survey that assesses perceived risk and related psychosocial measures.

### Intervention and control procedures

**Fig. 1:**
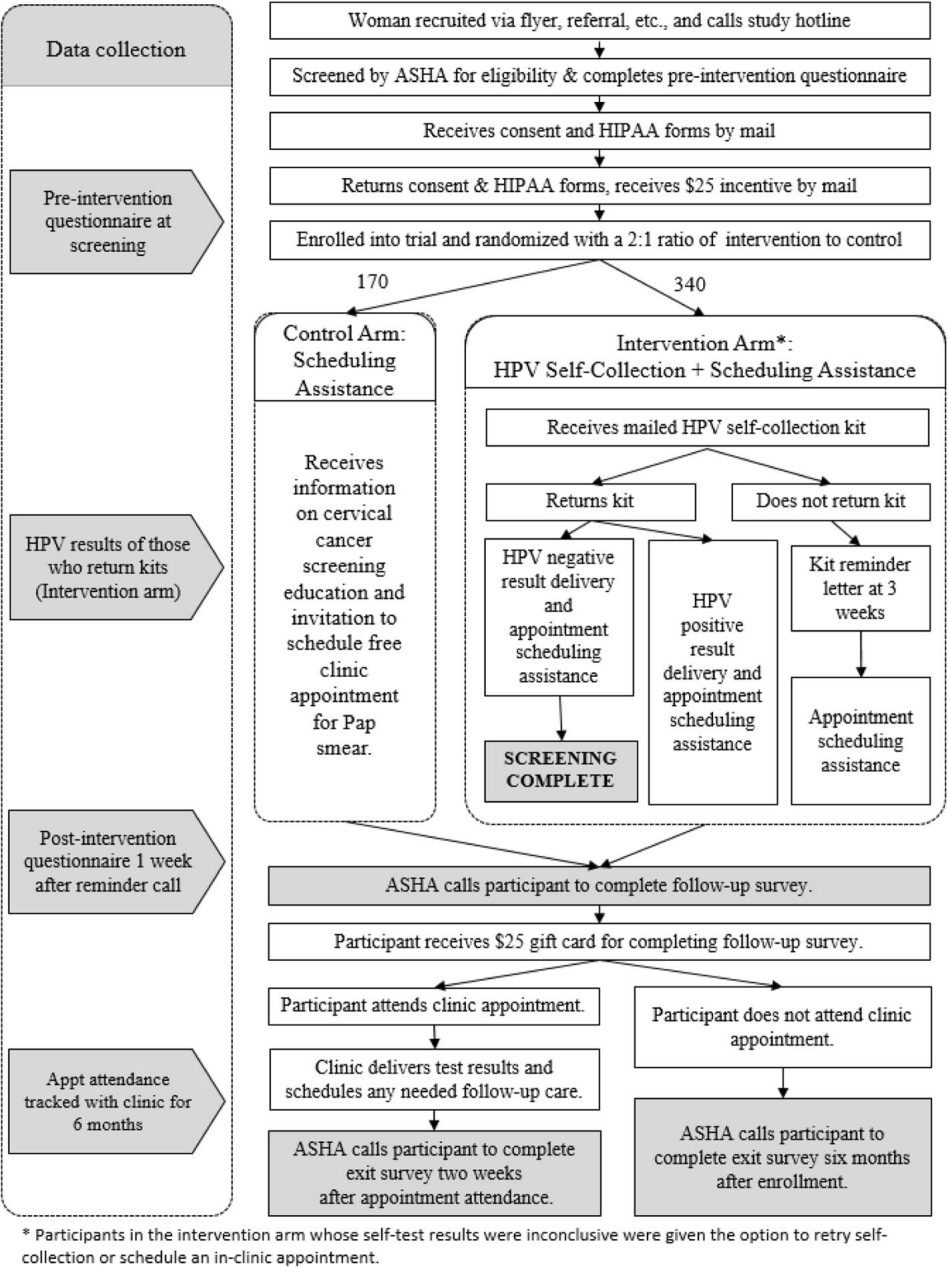
MBMT study flow. ASHA, American Sexual Health Association; HPV, Human papillomavirus; HIPAA, Health Insurance Portability and Accountability Act

Women assigned to the intervention arm will receive an HPV self-collection kit by mail, which will include an informational sheet, instructions, and materials to self-collect a cervico-vaginal sample, and a pre-paid envelope to return it by mail (Fig. 1). When HPV self-collection test results are available, ASHA staff will call the participant to deliver her HPV results and schedule her for a free appointment in clinic regardless of the result. Women who with self-collection HPV-positive results will be informed that their results indicate a higher risk for cervical cancer and will be advised to schedule an appointment to complete Pap/HPV co-testing at a study clinic (see results delivery scripts, Additional file 1: Appendix 1). Women with self-collection HPV-negative results will be told that their results indicate lower risk for cervical cancer, but that doctors cannot use self-collection results to make decisions about their care. Participants with both HPV-negative and HPV-positive self-collection results will be able to schedule an appointment for free screening in clinic during the HPV results delivery call. Participants whose self-collection results are inconclusive will be contacted and given the option to try the self-collection again or schedule a clinic appointment. A participant who has not returned a self-collected sample within 3 weeks will receive a mailed reminder letter to return the kit. If the kit still has not been returned 2 weeks later, she will receive a reminder call, at which point she will receive assistance to schedule a clinic appointment if she prefers.

Control-arm participants will receive scheduling assistance 1 week after enrollment. This scheduling assistance call provides brief education on cervical cancer and screening, followed by the opportunity to schedule a free screening appointment in clinic for Pap/HPV co-testing. Education will emphasize that cervical cancer is preventable with regular screening and early treatment; the participant is due for screening based on doctor recommendations; and the participant is eligible for free screening at a local clinic. Participants will be directed to go to a study-associated clinic based on residence.

Up to three call attempts will be made to contact a participant to deliver her self-test results or to assist with scheduling a clinic appointment. If these call attempts fail, the study will send a final letter inviting the participant to call the study hotline to receive her HPV self-test results. Participants with HPV self-test positive results who are not reached by phone will receive a letter with their results, strongly recommending screening in clinic.

### Sample collection and laboratory testing procedures

The self-collection kit will contain a brush and vial of sample preservation solution, simple illustrated instructions, the study hotline to call with questions, and a pre-addressed, pre-paid mailer for sample return. An information sheet will also be included in the kit to provide education about HPV, cervical cancer, and screening by clinic and self-collection methods. As in previous studies [42, 47–49], sample self-collection will be performed using a Viba brush (Rovers Medical Devices, B.V., The Netherlands), which the participant will insert into the vagina as far as the brush can comfortably go, and she will rotate it five times and then remove it. The participant will then separate the brush head from the handle using a small brush remover tube and place it into a collection tube containing 4.3 mL of Aptima sample transport medium (Hologic, Inc., San Diego, CA, USA). The sample transport medium is non-toxic, approved for mailing via the US Postal Service and validated to keep specimens stable for HPV RNA testing for at least 60 days at room temperature. We developed the educational materials, scripts, and illustrated instructions for completing self-collection in English and Spanish for low-literacy populations using National Institutes of Health (NIH) plain language guidelines [50] and based on pilot-testing the materials with women in the target population. Participants who attend an appointment at a study clinic will receive a pelvic exam by a clinical professional to collect a cervical sample using an endocervical brush and spatula, which the clinician will place immediately into PreservCyt media (Hologic, Inc.).

Samples self-collected at home will be sent to study staff and then for testing at the Laboratory Corporation of America laboratory in Burlington, North Carolina. Clinician-collected samples will be sent directly to the laboratory for testing. The laboratory will perform HPV testing on self-collected and clinician-collected samples using the Aptima HPV assay (Hologic, Inc.), an FDA-approved molecular amplification assay that qualitatively detects E6/E7 mRNA of 14 HPV genotypes (16, 18, 31, 33, 35, 39, 45, 51, 52, 56, 58, 59, 66, and 68), according to the manufacturer’s instructions [51]. For samples testing positive for the high-risk HPV panel, the laboratory will test for HPV types 16 and 18/45 using the FDA-approved Aptima 16 18/45 assay. All Aptima assays and instrument systems are FDA-approved for testing clinician-collected specimens. Though HPV testing on samples self-collected at home is not FDA-approved for any assay, studies have validated the Aptima HPV assay for self-collection [47, 48, 52–54]. To determine co-infection with other sexually transmitted infections, self-collected and clinician-collected samples will be also tested for *Chlamydia trachomatis* and *Nesseiria gonorrheae* using the Aptima Combo2 assay and for *Trichomonas vaginalis* using the Aptima TV assay (all Hologic, Inc.). A trained operator at the laboratory will perform these tests on the fully automated Panther system, according to the manufacturer’s instructions; clinician-collected samples will be also analyzed for liquid-based cytology using the ThinPrep 2000 Processor (Hologic, Inc.) and classified according to the 2001 Bethesda System at the Labcorp laboratory. If any cytological cervical abnormality (i.e., atypical squamous cells of undetermined significance or worse), HPV, or sexually transmitted infection is identified in a clinician-collected cervical sample, the clinician will refer the participant for follow-up diagnostics and treatment per standard-of-care [55].

### Data collection and measures

Participants will complete three questionnaires: a baseline questionnaire at enrollment, a follow-up questionnaire 1 week after self-collection result delivery or appointment scheduling call, and an exit questionnaire 2 weeks after the participant attends a screening appointment or 6 months after enrollment for participants who do not attend a screening appointment. Data collected on each questionnaire are shown in Table 1. ASHA call-center staff will administer participant questionnaires over the phone in English or Spanish. Regardless of whether participants complete screening, they will receive US$25 each for the baseline and follow-up questionnaires, and US$30 for the exit questionnaire. Given that compensation is linked only to questionnaires and not to self-collection completion or clinic attendance, screening completion should not be affected by financial motivations.

**Table 1:** Questionnaire data collected in the My Body My Test-3 study

	Survey Timing		
	Baseline	Post-intervention	Exit
Sociodemographic data	X		
Knowledge about cervical cancer and screening	X	X	
Perceived likelihood of cervical cancer	X	X	
Worry about getting cervical cancer	X	X	
Embodiment of risk	X	X	
Perceived barriers	X	X	
Self-efficacy	X	X	
Acceptability of and attitudes towards self-collection		X	
Acceptability of and attitudes towards in-clinic screening			X
Patient costs associated with in-clinic screening			X

#### Primary outcome

The primary trial outcome will be completion of cervical cancer screening within 6 months of study enrollment. Screening completion will be defined as screening in clinic or receipt of negative HPV self-collection results. Women with HPV negative self-collection results will be considered screening-complete. Control-arm participants and women with HPV-positive self-collection results will be considered screening-complete if they obtain co-testing or Pap test screening at a study-affiliated or other clinic. Study-associated clinics will report participant clinic attendance directly to the study. Participants who have not attended an appointment at a study clinic will be asked at their 6-month exit questionnaire if they obtained screening at another clinic. If we are unable to reach the participant by phone for the exit questionnaire, we will send emails, texts, and finally a letter to request confirmation whether they attended an in-clinic appointment. If these attempts fail, we will contact other clinics in the participant’s area of residence to determine if the participant attended a screening appointment at another location during these 6 months. Women who undergo screening at any clinic, regardless of whether it is a study clinic, will be considered as having completed the primary endpoint.

#### Secondary outcomes and other measures

**Fig. 2:**
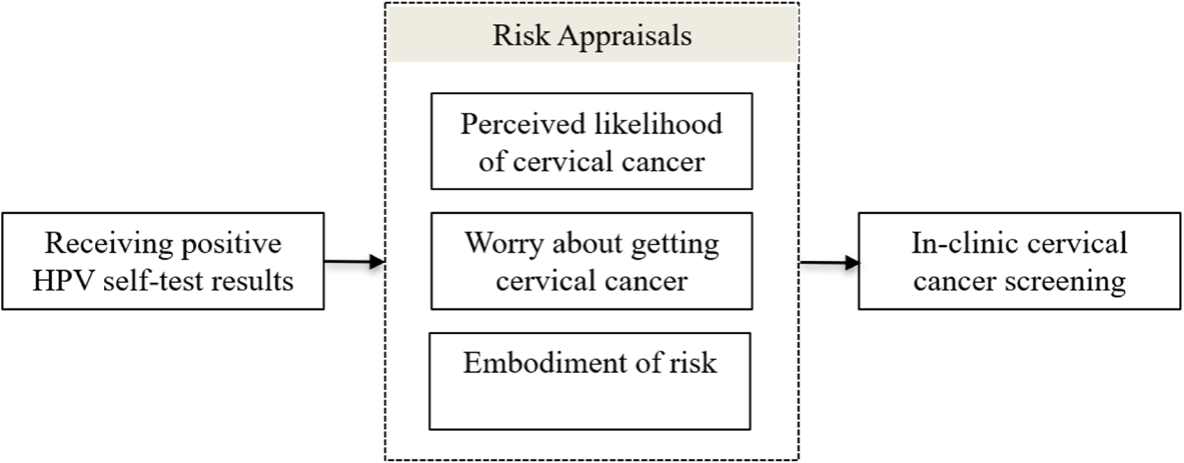
MBMT model adapted from the health belief model. HPV, Human papillomavirus

Secondary trial outcomes include perceived likelihood of cervical cancer, worry about getting cervical cancer, and embodiment of risk (Fig. 2). These constructs come from the health belief model (HBM) [33], which posits that an increase in the perceived risk of cancer will be associated with increased intention and subsequent completion of cancer screening [32, 56]. Perceived risk has a cognitive aspect, operationalized as *perceived likelihood* of developing cervical cancer, and an affective aspect, operationalized as *worry*. For the present trial, we added an exploratory risk construct, *embodiment of risk,* which is similar to experiential risk in the TRIRISK model, which we operationalize as awareness of and a sense of connection with one’s body as a source of health and risk [57, 58].

We will collect data on costs associated with each arm to estimate the incremental cost per woman screened in the intervention arm as compared to the control arm. Material costs will include those related to training, recruitment, postage, self-collection kit processing, and laboratory testing. Personnel costs will include time spent on staff training, recruitment, mailings, scheduling clinic appointments, and results delivery (Table 2). The study database will automatically generate time stamps to record the time spent by call-center agents on phone calls for self-collection results delivery and appointment scheduling and on failed attempts to complete those calls. Personnel costs will be calculated by total time spent on an activity and the average hourly wage of the agent/staff member completing the activity. Research-specific costs will not be included, such as participant incentives or time spent delivering questionnaires.

**Table 2:** Cost data collected in the My Body My Test-3 study

	Cost Perspective	
	Health clinic	Public payers
Training		
Developing and delivering call-center training, study personnel time		X
Training call-center agents, materials		X
Attending call-center training, agent personnel time		X
Recruitment		
Recruitment materials and advertisements^a^		X
Recruitment personnel time^b^		X
Screening identification for eligibility		
Screening calls, personnel time		X
Self-collection for HPV testing		
Non-personnel costs		
Self-collection kits, materials, and postage		X
Processing self-collection kits, laboratory materials		X
Reminder letters, as needed, materials and postage		X
Personnel time costs		
Mailing self-collection kits		X
Delivering self-collection results and scheduling clinic appointments		X
Mailing reminder letters, as needed, materials		X
Clinic services and procedures		
Non-personnel costs		
Processing screening tests, laboratory materials	X	
Personnel time costs		
Scheduling appointment with patient for in-clinic co-testing	X	
Processing/completing paperwork for new patient	X	
Checking patient in and out	X	
Contacting patient who missed appointment	X	
Reporting abnormal screening results to patient and making referral for follow up	X	
Reporting normal screening results to patient	X	
Cost/cost recovery for procedures		
Medicaid reimbursement for screening test	X	X

*ASHA* American Sexual Health Association, *HPV* Human papillomavirus

^a^ Costs include postcards, flyers, yard signs, logo design, newspaper ads, radio station advertisements, website, transportation advertisements, and others

^b^ Costs related to developing and maintaining community partner relationships, direct outreach and recruitment events, distributing recruitment materials, posting advertisements, travel, and administrative procedures

#### Data management and monitoring

All data will be stored in a secure database accessible only to approved study staff. Call-center agents will enter questionnaire data directly into the database during study interviews. Regular checks will be conducted on questionnaire data to identify issues such as inadequate detail obtained from open-ended questions, lack of variation in responses, or unexpected response distribution that might indicate systematic error in data entry. Self-collection and clinic laboratory results will be delivered to study personnel via the Beacon secure results portal and entered by study personnel into the secure database.

#### Confidentiality

Consent forms, HIPAA authorizations, and other paper materials that contain participants’ identifying information will be stored in a locked cabinet accessible only to authorized study personnel. All electronic data will be stored in a secured database and managed by the University of North Carolina (UNC) information technology personnel. All paper records will be destroyed 7 years after the completion of the study. As outlined on the informed consent form (see Additional file 1: Appendix 2), self-collected and clinician-collected samples will be stored de-identified for possible future research.

### Analytic approach and power

Key demographic characteristics (e.g., age, race, marital status, education, income, years since last screening, and travel time between home and clinic) will be compared between study arms to assess any chance imbalances in covariates during randomization. Continuous variables will be analyzed using the Wilcoxon rank sum test, and categorical variables using Fisher’s exact test.

Primary analyses will compare the primary study outcome of screening completion between the study arms, employing the intent-to-treat (ITT) principle, wherein outcomes are analyzed according to study arm regardless of whether the participants complied with the components of the study arm intervention. Using log-binomial regression, the Z statistic for comparison of two binomial proportions will be used to compare the proportion of women in each arm that completed cervical cancer screening and will be reported with 95% confidence intervals based on the normal approximation to the binomial. While we do not expect any baseline covariates to be associated with randomization, we will adjust for any covariates that are associated with randomization and with completion of screening. Such adjustment will account for unexpected chance imbalances in covariates between the trial arms and potentially improve efficiency of inference about the primary outcome. Additionally, to account for potential differential drop-out rates between the two study arms, we will use inverse probability of censoring weights.

Secondary analyses will include per-protocol analyses, where we will assess the effect of the intervention among the subset of study participants who were compliant with their randomization assignment and the study protocol. Exploratory analyses will compare positive HPV detection rates, CIN2+ detection rates, self-collection/clinic-based screening, Pap test participation, follow-up colposcopy and treatment (if indicated), and differential intervention effects by race, age, income, and education level, between the two study arms.

To examine possible mechanisms explaining the intervention effect, a standard mediation analysis will be performed by (1) establishing whether the exposure of interest (receipt of HPV-positive result via self-collection) is associated with the outcome (screening in clinic) and mediating variables (e.g., perceived risk), and then (2) determining whether controlling for the mediators meaningfully reduces the size of the association between the exposure of interest and outcome. We will compare completion of screening in clinic in women in the intervention arm who return a self-collected sample and receive a positive HPV self-collection result and in (i) women in the intervention arm who do *not return* a self-collection sample (“non-returners”) and (ii) all women in the control arm, using exact logistic regression (adjusting for baseline covariates and potential confounders). HBM constructs will be evaluated as potential mediators of any observed association. The MacKinnon method will be used to compare coefficients for the intervention-behavior pathway, before and after controlling for the mediator, using the Sobel test [59, 60]. Appropriate methods for handling missing data (e.g., baseline value carried forward or multiple imputation) will be employed to account for unobserved mediator variables. If the intervention is not found to be effective, we will still conduct the mediation analysis to examine suppression (i.e., if the intervention increases one belief but reduces another, these changes could offset one another in how they affect behavior).

Primary cost assessment will take the perspective of healthcare providers and public payers, who acquire many of the cancer-related screening costs for uninsured and Medicaid-insured women. Costs and a surrogate marker of cervical cancer prevention effectiveness (i.e., women that complete screening) will be measured. The effectiveness measure will have a range of potential values drawn from the trial results, defined by a beta distribution, with costs for each arm defined by a distribution appropriate to the shape of the data. Using Monte Carlo simulation with 10,000 iterations, joint distributions of costs and effectiveness will be estimated for each arm, as well as the proportion of iterations for which HPV self-collection produces the highest net monetary benefit at varying levels of willingness to pay (WTP) [61–63]. Net monetary benefit is defined by the equation (Effectiveness x WTP) - Costs, where the optimal choice will be the intervention that has the highest net monetary benefit at a given value for WTP. For example, at a WTP of US$0, the least expensive option will always be favored, whereas at very high values of WTP, the most effective option will be favored. Given that there is no commonly accepted WTP [64], we will report the net monetary benefit at different potential values of WTP, which will allow decision makers to determine whether to implement self-collection of samples at home for HPV testing in their settings based on their own WTP per woman screened.

#### Power calculations

A total of 510 participants in a 2:1 randomization ratio of intervention to control provides 88–94% power to detect a 15% or greater difference between arms for the primary outcome, assuming intervention-arm screening completion of 60–80%, which would be consistent with previous studies [47, 65]. A rate of 13% HPV messenger RNA (Mrna) positivity (as detected in MBMT2 using the Aptima HPV assay in the same target population [47]) and a 70% return of kits in the intervention arm will provide 80–85% power to detect a 30% difference in uptake of clinic screening between participants with self-collection HPV-positive results and other subgroups for mediation analysis.

### Dissemination

Findings will be shared through scientific publications, participation at scientific meetings, and other public venues. Results will be made available on ClinicalTrials.gov, and the final dataset will be archived in a recommended data repository per NIH Data Sharing Policy Guidance and Implementation Guidelines.

## Discussion

This trial will help fill gaps in our understanding of the effect of self-collection of samples at home for HPV testing in increasing cervical cancer screening rates among low-income, hard-to-reach women at highest risk of cervical cancer in the USA. Though studies in other countries have shown that offering cervical cancer screening by HPV testing through mailing samples self-collected at home is effective at increasing cervical cancer screening uptake among under-screened women, this approach has not been evaluated in the context of the US healthcare system. Performance in the USA may differ from that in other countries, given the unique cultural context and lack of national screening registries or universal health care.

Other RCTs have been conducted in the USA to examine the effectiveness of HPV testing by self-collection delivered via mail and via community health worker, compared to screening in clinic [43, 66, 67]. Our study differs from these previous studies in that they defined their primary outcome as the number of women who attended a clinic screening appointment or returned a self-collection kit. In other words, participants who received a positive HPV self-collection result were considered as screening-complete. In contrast, in our study, a woman who receives a positive HPV self-test result will not be considered screening-complete until she has also attended a screening appointment in clinic. In addition to presenting this primary outcome, we will present our self-collection/clinic-based screening participation rate as an exploratory analysis to allow comparison to the primary outcomes of these previous studies.

This trial has two additional major strengths. First, it will advance scientific knowledge of psychological factors that mediate the relationship between HPV self-collection and subsequent screening in clinic. Assessment of these psychological factors will provide insight into possible ways to maximize the HPV self-collection intervention effect. Second, providing detailed, relative-cost data along with findings on clinical performance will give important information to state and national decision-makers to decide whether to implement HPV self-collection in their services offered, should it prove to be effective. These data will be particularly useful in fixed-resource settings, such as those serving uninsured and Medicaid-insured women. Because state and safety net health budgets are usually limited, assessing monetary value is critical.

Our RCT does have some limitations that should be noted. First, our sample may not be representative of the total population of under-screened women, since some high-risk women may not respond to printed materials or online or radio advertisements, our primary mode of recruitment. However, we will also directly recruit individuals through community events and organizations. Second, while women are randomized to a trial arm, participants themselves are not blinded to their randomization assignment. It is possible that individuals in the control arm may forgo screening due to desire to be in the intervention arm and vice versa.

HPV self-collection has the potential for widespread impact on public health because an increase in screening completion would have a direct effect on cervical cancer incidence and mortality. If self-collection of samples at home is found to be effective in increasing cervical cancer screening completion among under-screened US women at reasonable levels of WTP and is found to be cost effective, there are several pathways for its future implementation. Healthcare providers and systems could undertake outreach efforts to distribute kits to their under-screened patients, similar to the current use of fecal immunochemical tests to increase colorectal cancer screening. Kits could be distributed using approaches such as (1) direct mailings based on medical records review, (2) clinics that do not traditionally provide cervical cancer screening (e.g., county STI clinics or seasonal flu clinics), or (3) public outreach events [68, 69]. There is also potential for phone-based dissemination of the self-collection kits. Each year, state and national surveys identify large numbers of women overdue for screening by national guidelines, yet these surveys are not followed up with action such as offering screening to these women. Potentially, mailing self-collection kits to these women may be a viable solution. Finally, hotlines such as the United Way 2–1-1 social assistance hotlines could offer kits to under-screened callers. Overall, these findings may inform future research and policy questions related to cancer screening access for hard-to-reach populations.

### Trial status

The trial was registered on ClinicalTrials.gov on 11 January 2016 as protocol NCT02651883. Recruitment began in April 2016, is currently ongoing, and will continue at least through December 2019.

## Supplementary information

**Additional file 1: Appendix 1.** Self-test results delivery and screening invitation: intervention group script. **Appendix 2.** Consent form.

## Data Availability

Investigators interested in using these samples or accessing the final dataset for future research may do so under the following conditions: (1) IRB approval has been obtained from the institution covering the investigator, (2) data security procedures ensuring patient privacy have been demonstrated by the investigator, and (3) a data use agreement is completed by UNC and the outside investigator. Final datasets for analysis will not include any identifying information.

## Abbreviations

ASHA: American Sexual Health Association
CIN-2+: Cervical intraepithelial neoplasia lesions grade 2 or higher
CPSTF: Community Preventive Services Task Force
FDA: Food and Drug Administration
FPL: Federal poverty level
HBM: Health belief model
HIPAA: Health Insurance Portability and Accountability Act
HPV: Human papillomavirus
MBMT: My Body My Test
NC BCCCP: North Carolina Breast and Cervical Cancer Control Program
NIH: National Institutes of Health
Pap test: Papanicolaou test
PI: Principal investigator
UNC: University of North Caroline
WTP: Willingness to pay
