# Supplementary material for: Impact of human papillomavirus (HPV) self-collection on subsequent cervical cancer screening completion among under-screened US women: MyBodyMyTest-3 protocol for a randomized controlled trial
Source: Trials. 2019 Dec 27;20:788. doi: 10.1186/s13063-019-3959-2 (PMC6935089; doi:10.1186/s13063-019-3959-2)
Supplement: Supplementary file 1 — Additional file 1: Appendix 1. Self-test results delivery and screening invitation: intervention group script. Appendix 2. Consent form. [file 13063_2019_3959_MOESM1_ESM.docx]

**Appendix 1: Self-test results delivery and screening invitation: Intervention group script**

Good morning/afternoon, this is __ and I’m calling from the UNC women’s health screening study, My Body My Test.

I’m calling because the results from your self-test are ready. Is this a good time to talk?

*Yes: Continue*

*No:* When would be a good time to call you back? *(Complete callback request)*

**Positive HPV result**

Your test was **positive** for HPV. This means that you **have** an HPV infection.

*Pause a moment to allow participant to process information.*

Remember that HPV is very common. Most women who have HPV stay healthy.

However, this HPV result is a warning sign. It means that you are at a higher risk for cervical cancer. It is very important that you get a Pap smear at a clinic as soon as possible.

Do you have any questions about these test results?

*[Answer as needed.]*

HPV tests and Pap smears are two different ways to look for cervical cancer. An HPV test, like the one you took, looks for the virus that can cause cervical cancer. A Pap smear looks for unhealthy or abnormal cells that might turn into or might be cancer. Doing a Pap smear and HPV test in the clinic will help your doctor decide whether you need more tests or treatment.

Remember that getting a Pap smear can prevent cancer. If a Pap smear finds a problem, doctors can usually do a simple treatment in the office to treat the problem before it turns into cancer. If your in-clinic screening finds a problem, we will help you get affordable treatment.

You are eligible for free cervical cancer screening at [load clinic name]. Would you like to make an appointment now?

*Participant wants to schedule an appointment.*

*Participant wants to be contacted later for appointment scheduling. (Complete callback request form).*

*Participant does not want to schedule an appointment:* We strongly recommend that you get a Pap smear as soon as possible. May I ask you are planning to go to a different clinic?

*Yes:* Could you please tell me where you plan to go? [record name of clinic/doctor]

*No:* No problem. If you decide later that you want to schedule an appointment, you can call us back any time. Can I give you our phone number? *[877-698-3781]*

**Negative HPV result**

Your self-test result is **negative** for HPV. This means that you probably do **not** have an infection with the kind of HPV that can cause cervical cancer.

It is probably safe for you to wait 2 years before you get screened for cervical cancer again. But, it is very important that you keep getting screened in the future.

Do you have any questions about your test results?

[Answer as needed.]

I want to give you some quick information about cervical cancer screening.

[Load for women <30: Women who are 21 to 29 years old should get Pap screening every 3 years.]

Women over 30 can get screened for cervical cancer with a Pap smear or an HPV test.

A Pap smear looks for unhealthy or abnormal cells that could turn into cervical cancer. An HPV test looks for a virus called human papillomavirus. This is the virus that can cause cervical cancer.

You can get a Pap smear by itself every 3 years. Or, you can get a Pap smear and an HPV test together every 5 years. Women might need to get screened more often if they have had an abnormal Pap result or have HIV.

Doing a Pap smear and HPV test together is best. If you are 30 years or older, the next time you get a Pap, ask your doctor to do an HPV test, too.

Your self-test showed that you probably do not have an HPV infection. Studies have shown that the self-test is very accurate, but no test is perfect. Doctors can’t use the results from the self-test to make decisions about your care, so if you want to confirm your results, you can schedule a free in-clinic screening at [load clinic name]. If you choose to schedule an appointment with us, you will get free Pap and HPV testing at that visit.

Would you like to make an appointment now?

*Do not let participant off the phone until STI results are delivered on next page.*

*Participant wants to schedule an appointment.*

*Participant wants to be contacted later for appointment scheduling. (Complete callback request form).*

*Participant does not want to schedule an appointment:* OK, could you tell me if you're planning to go to a different clinic?

*If yes:* Could you please tell me where you plan to go? [record name of clinic/doctor]

*No:* No problem. If you decide later that you want to schedule an appointment, you can call us back any time. Can I give you our phone number? *[877-698-3781]*

**Inconclusive HPV result**

Your self-test did not get a clear result for whether or not you have HPV infection. This means we can’t tell if you have HPV or not. This happens sometimes, and it doesn’t mean there’s anything wrong.

Because you are overdue for cervical cancer screening, we recommend you do another test as soon as possible. I’d like to give you some quick information about screening. It is important to get screened regularly, because screening can actually **prevent** cervical cancer. If a screening test finds a problem, doctors can usually do a simple treatment in the office to stop the problem before it turns into cancer.

[Load for women <30: Women who are 21 to 29 years old should get Pap smears every 3 years.]

Women over 30 can get screened for cervical cancer with a Pap smear or an HPV test.

A Pap smear looks for unhealthy or abnormal cells that could be cervical cancer, or could turn into cervical cancer. An HPV test looks for a virus called human papillomavirus. This is the virus that can cause cervical cancer.

You can get a Pap smear by itself every 3 years. Or, you can get a Pap smear and an HPV test together every 5 years. Women might need to get screened more often if they have had an abnormal Pap result or have HIV.

Doing a Pap smear and HPV test together is best. If you are 30 years or older, the next time you get a Pap, ask your doctor to do an HPV test, too.

You can also test for HPV first using the self-test we sent you. This test will tell you about your risk for cervical cancer, which can help you decide whether you want to go to a clinic for additional testing. Or, I can schedule an appointment now for you to get free Pap and HPV testing at [load clinic name]

Would you like to do another free self-test by mail, or would you prefer to make an appointment to get free screening at [load clinic name]?

***Do not let participant get off phone until STI results are delivered on next page.***

*[Response options are as below, with the additional option of…]*

*Wants to do a self-test:* Great, can I please confirm your mailing address? *[Address loads to view/ edit]:* We will send another kit. You should get it in the next week.

**Appendix 2: Consent Form**

**University of North Carolina at Chapel Hill**
**Consent to Participate in a Research Study**

**Title of Study**: My Body, My Test 3 – Effect of HPV Self-Collection on Cervical Cancer Screening in High Risk Women
**Person in charge of this study**: REMOVED
**Where she works**: REMOVED
**Her phone number**: REMOVED
**Her email address**: [REMOVED](mailto:jsssmith@email.unc.edu)

- This form has information about being in our research study.
- You should sign and return this form if you want to be in the study.
- You do not have to be in the study.
- You can choose to quit the study at any time.
- Please read this information carefully.
- Please call us if you have any questions: (919) 966-6766

**Who is paying for this study:**
National Institutes of Health (NIH), National Cancer Institute (NCI)

**Study Contact Telephone Number**: (919) 966-6766
**Study Contact Email**: mybodymytest@unc.edu

**Consent Form Version Date:** March 21, 2018
**IRB Study #** 14-3042

**Why are we doing this study?**

We want to find easier ways to help get women screened for cervical cancer. We are comparing two ways for women to get screened: helping them schedule an appointment for free screening at a doctor’s office, or offering them a self-test to complete at home. We are asking you to join the study because you have not had a Pap smear recently.

**What are some general things you should know about research studies?**

Research studies are designed to find out new things that might help other people. Being in the study might not help you, personally. There also might be some risks to being in a research study.

You do not have to be in this study if you don’t want to. Even if you say yes now, you can stop later if you want to. If you say no, it will not affect your health care or any other service you get.

Below there are details about this study. It is important that you understand this information. You can call us at (919) 966-6766 or email us at mybodymytest@unc.edu if you have any questions. You will be given a copy of this consent form to keep.

**Are there any reasons you should not be in this study?**

You should NOT be in this study if…

- You are younger than 25 or older than 64
- You are pregnant
- You had a Pap smear in the last 3 years
- You have had a complete hysterectomy (removed all of the uterus)

**How many people will be in this study?**

We will enroll about 600 women.

**How long will your part in this research study take?**

The study will take about 1 hour of your time over about a month:

- You have already done a phone survey, which may have taken 20 minutes.
- It will take about 10-15 minutes to read, sign, and send back this consent form, the HIPAA authorization form, and the contact information form in this packet.
- It will take 15-20 minutes to do a second phone survey.
- It will take 5-10 minutes to do a third phone survey.

Cervical cancer screening will take additional time if you choose to get screened.

**What will happen if you decide to be in the study?**

If you join this study, we will put you into one of two groups: women who will have the opportunity to schedule a free Pap smear appointment, or women who have the opportunity to try the self-test and/or to schedule a free Pap smear appointment. You will be randomly picked to be in one of these groups (picking randomly is like flipping a coin). If you sign up for the study, you need to be ok with the possibility that you could be in either group. We can’t pick or change your group.

If you agree to be in this study, you will need to:

1. Sign and return this consent form, the HIPAA consent form, and the contact form.
2. Complete two more surveys by phone.

Everyone in the study will have the option to get free cervical cancer screening if they want to. We encourage you to get screened to protect your own health, but you do not have to do it and you will not get paid for doing it. You will not have to pay for any of the tests or appointments you choose to do as part of this study.

If you do the self-test and/or go to the doctor for a Pap smear, we will test those samples for human papillomavirus (HPV), the virus that can cause cervical cancer. We will give you the results of these tests and make sure you understand what the results mean. We will then store these samples anonymously for future research.

**Is the self-test safe and accurate?**

Yes. The brush and liquid in the self-test kit have both been approved by the FDA as safe for self-testing. The liquid in the tube is non-toxic. Self-tests for HPV are almost as accurate as HPV tests done by a doctor, and they are more likely to find disease than Pap smears. However, the HPV self-test is not approved or cleared for clinical use by the FDA, and LabCorp has not confirmed the performance standards of the test. This means that your doctor can’t use the results from your HPV self-test to make decisions about follow-up screening or treatment. Your doctor will use the results from the Pap smear, if you get one, to decide if you need to get follow-up testing or treatment.

**What will we do with your samples when this study is over?**

We will store your samples for future research that might help us answer important questions like how cervical cancer develops and what other organisms are found with HPV. Samples will be labeled with a study number and stored in a freezer. The study number will not be connected to any names or other private information**.** We will not share any samples with a researcher until they explain what it will be used for. We will not give names or private information to anyone.

Once the sample is stored, we can’t remove it from storage. It will be impossible to know whom the samples are from after they are stored because personal information will not be connected to any sample.

**What happens if you say no, you do not want to be in the study?**

That is totally ok. You will not lose any benefits and it will not change your relationship with your doctor or anyone else that provides services to you. You are most likely eligible to get a free or low-cost Pap smear at the health department or other doctors’ offices in your area without participating in the study.

**What happens if you say yes, but change your mind later?**

You can stop being in the study at any time. You will not be penalized. However, you will only get a gift card for each survey you complete.

**How will we keep information private?**

Only people who work on the study can see your survey answers. Only your doctor, the lab that does the tests, and the people who work on the study can know your test results. All of this information is stored in a secure database. Your consent forms will be stored in a locked filing cabinet accessible only by study staff. We are not allowed to share your information with anyone else unless you give us permission.

You will not be identified in any report or publication about this study. Although every effort will be made to keep research records private, there may be times when federal or state law requires the disclosure of such records, including personal information. This is very unlikely, but if disclosure is ever required, UNC-Chapel Hill will take steps allowable by law to protect the privacy of personal information. In some cases, the information in this research study could be reviewed by representatives of the University, research sponsors, or government agencies (for example, the FDA) for purposes such as quality control or safety.

**Will it cost you anything to be in the study?**

You might need to pay for minutes if you complete the phone surveys on your cell phone. The gift cards we will give you for doing the surveys will cover these costs. There should not be any other costs for doing study activities. If you choose to go to the doctor to get a Pap smear, you might have to pay for transportation, parking, or childcare. You will not have to pay for the doctor visit or laboratory tests. If your tests find a problem that needs further testing or treatment, we will help you find affordable care.

**Will being in this study help you in any way?**

You will get:

- Information about cervical cancer and screening.
- Help getting free cervical cancer screening, if you want it.

As an additional service to protect your health, we can test your samples for gonorrhea, chlamydia, and another common infection called trichomonas vaginalis. If you go to the doctor’s office and your test shows that you have gonorrhea or chlamydia, your doctor is required to report it confidentially to a state agency that tracks these diseases. They will help you get free treatment. We encourage you to get these tests, but you can choose not to. Please check this box if you do NOT want this testing.

🞏 NO, do NOT test my samples for chlamydia, gonorrhea, and trichomonas vaginalis.

**Will you be paid for your time?**

Yes. We will mail you a $25 gift card when you send back the forms in this packet. We will mail you another $25 gift card when you do the second phone survey in a few weeks. We will mail you another $30 gift card when you do the third survey in a few months. If you complete all 3 surveys, we will also enter you in a drawing to win a $200 Visa gift card.

**Are there any risks to you for being in this study?**

It is possible that:

- The survey questions might make you embarrassed or sad. You can skip any questions you want to. We will be very careful to keep your information private.
- You might feel uncomfortable while doing the self-test or getting a Pap smear. Some people might feel pain and/or experience bleeding, which should not be heavy. The discomfort and bleeding should only be temporary. Providers performing the exam are trained and certified
- Your test results might make you worried or upset. We will explain your results to you and can give you more information and counsel you if needed.
- Someone could find out that you were in the study and learn something about you that you did not want them to know. This is very unlikely, because we will do everything we can keep your information private.

**What if we learn about new findings or information during the study?**

We will tell you if we learn anything during the study that might affect your willingness to continue. If you want, we can also tell you about what we learn at the end of the study – just let us know by checking the box at the end of the form.

**Who is paying for this study?**

This research is paid for by the National Cancer Institute (NCI), which is part of the National Institutes of Health (NIH). This means that they are paying the research team to do the study. The researchers do not, however, have a direct financial interest with the sponsor or in the final results of the study.

**What if you have questions about the study?**

Please call the project coordinator, REMOVED, (919) 966-6766 or email mybodymytest@unc.edu if you:

- Have any questions about the study.
- Have questions about your rights.
- Feel you have been hurt in any way by being in this study.

**What if you have questions about your rights as a research participant?**

All research on human volunteers is reviewed by a committee that works to protect your rights and welfare. If you have questions or concerns about your rights as a research subject, or if you would like to get information or give feedback, you may contact the Institutional Review Board at 919-966-3113 or by email to IRB_subjects@unc.edu.

**Participant’s Agreement**:

**1. Other Research Studies** (please check one)

We might want to contact you in the future about other research studies.

- I agree to be contacted about other research studies.

OR

- I do not want to be contacted about other research studies.

**2. Results of the study**

If you want, we can send you a letter when we’re done with the study to tell you what we learned.

🞏 When the study is over, I would like to receive a letter telling me what was learned.

I have read the information provided above.  I have asked all the questions I have at this time.  I voluntarily agree to participate in this research study.

| ______________________________________________________ Your Signature | ____________________ Date |
| --- | --- |
| ______________________________________________________ Your Name (please print) |  |
